# Characterizing the ribosomal tandem repeat and its utility as a DNA barcode in lichen-forming fungi

**DOI:** 10.1186/s12862-019-1571-4

**Published:** 2020-01-06

**Authors:** Michael Bradshaw, Felix Grewe, Anne Thomas, Cody H. Harrison, Hanna Lindgren, Lucia Muggia, Larry L. St. Clair, H. Thorsten Lumbsch, Steven D. Leavitt

**Affiliations:** 1 Department of Biology, Brigham Young University, 4102 Life Science Building, Provo, UT 84602 USA; 2 Grainger Bioinformatics Center, The Field Museum, Chicago, IL USA; 3 Science & Education, The Field Museum, Chicago, IL USA; 4 Department of Life Sciences, University of Trieste, via Giorgieri 10, 34127 Trieste, Italy; 5 M. L. Bean Life Science Museum, Brigham Young University, 4102 Life Science Building, Provo, UT 84602 USA

**Keywords:** Copy number variation, DNA barcoding, ITS, Lichens, Repeat region, *Rhizoplaca*

## Abstract

**Background:**

Regions within the nuclear ribosomal operon are a major tool for inferring evolutionary relationships and investigating diversity in fungi. In spite of the prevalent use of ribosomal markers in fungal research, central features of nuclear ribosomal DNA (nrDNA) evolution are poorly characterized for fungi in general, including lichenized fungi. The internal transcribed spacer (ITS) region of the nrDNA has been adopted as the primary DNA barcode identification marker for fungi. However, little is known about intragenomic variation in the nrDNA in symbiotic fungi. In order to better understand evolution of nrDNA and the utility of the ITS region for barcode identification of lichen-forming fungal species, we generated nearly complete nuclear ribosomal operon sequences from nine species in the *Rhizoplaca melanophthalma* species complex using short reads from high-throughput sequencing.

**Results:**

We estimated copy numbers for the nrDNA operon, ranging from nine to 48 copies for members of this complex, and found low levels of intragenomic variation in the standard barcode region (ITS). Monophyly of currently described species in this complex was supported in phylogenetic inferences based on the ITS, 28S, intergenic spacer region, and some intronic regions, independently; however, a phylogenetic inference based on the 18S provided much lower resolution. Phylogenetic analysis of concatenated ITS and intergenic spacer sequence data generated from 496 specimens collected worldwide revealed previously unrecognized lineages in the nrDNA phylogeny.

**Conclusions:**

The results from our study support the general assumption that the ITS region of the nrDNA is an effective barcoding marker for fungi. For the *R. melanophthalma* group, the limited amount of potential intragenomic variability in the ITS region did not correspond to fixed diagnostic nucleotide position characters separating taxa within this species complex. Previously unrecognized lineages inferred from ITS sequence data may represent undescribed species-level lineages or reflect uncharacterized aspects of nrDNA evolution in the *R. melanophthalma* species complex.

## Background

For eukaryotes, regions within the nuclear ribosomal (nrDNA) operon have been instrumental in characterizing diversity and inferring evolutionary relationships [1, 2]. The eukaryotic nuclear ribosomal operon is arranged in tandem repeats in the nuclear genome, with each repeat containing genes, various spacer regions, introns, and other less understood elements [3–5]. Copy number of the nrDNA operon is a rapidly evolving trait [6]. Across Fungi, nrDNA copy number has been shown to vary considerably, ranging from tens to over 1400 copies per genome [7]. The relative ease of amplification, coupled with variable substitution rates among different regions of the nrDNA, have promoted its longstanding use in phylogenetic and biodiversity research [1]. Impetus for including the full nrDNA operon in published genome assemblies [8] and use of nrDNA in emerging long-read sequencing technologies [1] highlights the need of improved characterization of this important genomic region.

The internal transcribed spacer region – ITS: comprising ITS1, 5.8S, and ITS2 – within the nrDNA operon has been designated as the standard DNA barcoding region for Fungi [9]. The ITS barcode currently plays a fundamental role in characterizing fungal diversity [10]. However, the integrity of the ITS region for barcoding is questionable due to studies reporting conflicting results as to the levels of intragenomic variation found within the fungal nrDNA operon [8]. Some studies report that mutations and variation within the ITS region are relatively minor and of little practical consequence, attributing the consistency to concerted evolution [11]. Other studies, however, report significant amounts of variation, suggesting that evolution may not occur in a purely concerted manner [12]. Studies using traditional Sanger sequencing may effectively conceal potential intragenomic variation due to polymerase chain reaction (PCR) bias or dominating signal from the predominantly amplified copy [13]. Cloned sequencing studies have revealed the occurrence of intragenomic variation in nrDNA in multiple fungal lineages [8, 14]. In fact, multiple pyrosequencing-based studies suggest that distinct ITS copies are found in multiple ascomycete and basidiomycete lineages, although in a relatively low proportion of sampled lineages [15, 16].

Information from nrDNA has been fundamental in research into lichen-forming fungi [4, 17–20]. Although some studies have demonstrated limitations of the ITS barcoding for delimitation of species [21, 22], others highlight the potential of DNA barcoding studies to improve our ability to characterize diversity of lichen-forming fungi [23–27]. The full nrDNA operon, to our knowledge, has not yet been characterized for any of the lichenized fungi examined to date. Furthermore, despite suggestive evidence that some lineages of lichenized fungi harbor multiple distinct copies of the ITS region [28], intragenomic nrDNA variation has not yet been explicitly tested to our knowledge.

**Fig. 1:**
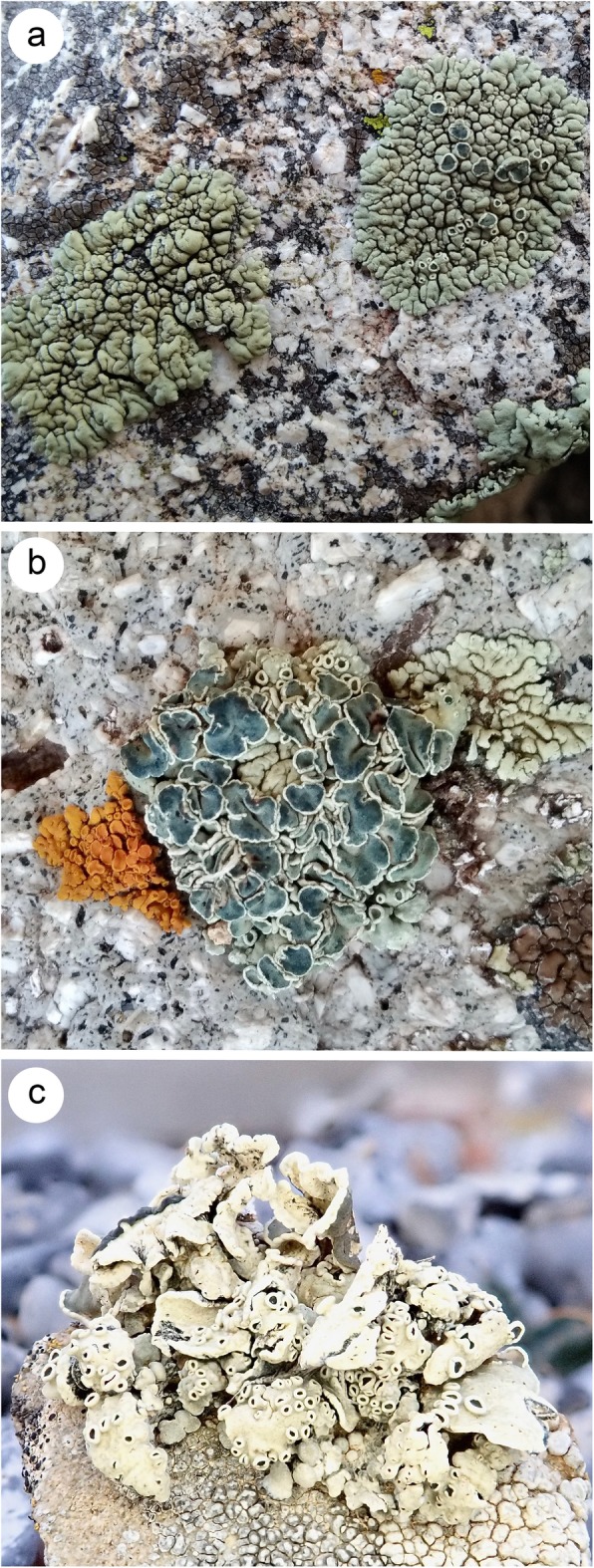
Representatives of the *Rhizoplaca melanophthalma* species complex. **a**., the crustose taxon *R. novomexicana* (field image, La Sal Mountains, Utah, USA); **b**., the umbilicate taxon *R. melanophthalma* sensu stricto (field image, La Sal Mountains, Utah, USA); and, **c**., the umbilicate taxon *R. porteri* (field image, Rich Co., Utah, USA)

In this study we investigated members of the *Rhizoplaca melanophthalma* species complex [29] (Fig. 1) with a goal of more fully characterizing features of nrDNA in a defined group of lichen-forming ascomycetes. The *Rhizoplaca melanophthalma* species complex is a monophyletic lineage currently consisting of approximately ten closely-related species/species-level lineages that originated during the Miocene and diversified largely during and Pleistocene [30]. Previous empirical species delimitation studies have circumscribed robust species boundaries among closely-related and morphologically similar species [29, 31], a pattern which has been supported by genome-scale molecular data [32, 33]. Formally described species in the complex can be identified using the standard DNA barcoding marker [31], although the taxonomy and evolutionary history of the vagrant species are more complex than previously considered [34].

To more fully characterize the nuclear ribosomal operon for the *Rhizoplaca melanophthalma* species complex, we (i) generated nearly complete assemblies of the nuclear ribosomal operon from short-read, high-throughput sequencing data, (ii) estimated the number of copies of the ribosomal operon repeat region, (iii) assessed the range of intragenomic variation in the ITS region – the formal DNA barcoding marker in fungi, and (iv) compared topologies inferred from different regions of the nuclear ribosomal operon. Furthermore, to better understand the range of nrDNA sequence variation in the *R. melanophthalma* species complex, we compiled and analyzed a nrDNA matrix comprised of the intergenic spacer region (IGS) and ITS sequences representing nearly 500 specimens. By characterizing nrDNA copy-number, assessing the potential intragenomic variation in the ITS region, phylogenetic resolution of different regions of the nrDNA, and overall genetic diversity, we aim to provide valuable insight into the utility of nrDNA operon as a barcoding marker for fungi.

## Results

Short reads are deposited in NCBI’s Sequence Read Archive under project PRJNA576709. Assembled sequences were submitted to GenBank: accession numbers MN756803– MN756835, complete nrDNA cistron sequences; and MN764257-MN764288, new ITS sequences generated via Sanger sequencing). Alignments generated and used in this study were submitted to TreeBase (https://treebase.org/; study No. 24225).

### Operon assemblies and coverage

In the initial SPAdes assemblies, the nrDNA operon was assembled into a single contig comprised of the complete 18S, ITS1, 5.8S, ITS2, and 28S regions, and most of the IGS region. However, in all specimens, a short region near the 5′ end of the IGS remained ambiguous due to a region of repeats expanding beyond the libraries’ insert size. Of the reads mapped to the initial nrDNA SPAdes assemblies, between ca. 85–92% were assembled as one contig representing the mycobiont nrDNA operon, 4–6% as contigs representing the photobiont nrDNA operon, and the remaining reads, ca. 3–7%, were assembled into small, low coverage contigs. Subsequent de novo Geneious assemblies using only reads initially mapped back to the original nrDNA contig from the SPAdes assembly were highly congruent with the original assemblies. For libraries run on the HiSeq platform, the average coverage of the nrDNA operon was 317x, ranging from ca. 79x (‘mela_8800’) to ca. 950x (poly_8668g). For the four specimens sequenced on the MiSeq platform, the average coverage of nrDNA was 1324x, ranging from ca. 376x (‘novo_8664d’) to ca. 1813x (‘subd_9052’) (Additional file 1). In all cases, the consensus ITS sequence from the nrDNA contig assembly was identical to the ITS sequences generated using Sanger sequencing.

In the *R. melanophthalma* group, the length of the nrDNA operon ranged from ca. 11.1–11.2 kb in *R. melanophthalma s. str.* to ca. 14.0–15.9 kb in *R. shushanii* (Table 1). In the outgroup taxa, *P. peltata* and *R. subdiscrepans*, nrDNA operons were considerably shorter – 8.5 kb and 8.9 kb, respectively. Differences in the lengths of the nrDNA operon were largely due to variable intron patterns in the 18S, 28S, and IGS regions. The aligned nrDNA operon (18S, ITS1, 5.8 S, ITS2, 28S, and IGS) included a total of 18,386 nucleotide position characters.

**Table 1:** Variation in the size of nrDNA operon

Taxon	Operon length^a^	Estimated copy #
*R. arbuscula* (2)	12.4 kb	14–21
*R. melanophthalma* (6)	11.1–11.2 kb	12–24
*R. novomexicana* (1)	12.4 kb	16
*R. occulta* (2)	12.6–12.7 kb	27–28
*R. parilis* (4)	12.6 kb	14–36
*R. polymorpha* (6)	12.9 kb	16–28
*R. porteri* (5)	11.7–11.8 kb	19–43
*R. shushanii* (5)	14.0–15.9 kb	9–22
*P. peltata* (1)	8.5 kb	13
*R. subdiscrepans* (1)	8.9 kb	11

^a^In all specimens, a short region near the 5′ end of the intergenic spacer region (IGS) remained ambiguous due to a region of repeats expanding beyond the libraries’ insert size and is not included in the total operon length

### nrDNA operon copy number estimates, intragenomic variation, and introns

We found low levels of putative intragenomic variation in the ITS region in members of the *R. melanophthalma* group, with variance at a single nucleotide position character rarely exceeding 10% (Additional file 2; Additional file 3). Furthermore, potentially polymorphic sites generally did not coincide with segregating sites that separated species (Additional file 3). The estimated copy number of the ribosomal operon ranged from 8.7 (standard deviation [SD] = 4.6) in *R. shushanii* (‘shus_8664–3’) to 42.7 (SD = 4.1) in *R. porteri* (‘port_8796’) (Table 1); and estimates were similar across a range of comparisons of nrDNA regions with single-copy regions of the nuclear genome (data not shown). Intraspecific nrDNA copy number variation was observed in all taxa represented by multiple individuals (Table 1). Furthermore, conspecific specimens collected at close spatial scales (< 100 m.) also showed variation in nrDNA copy number.

**Fig. 2:**
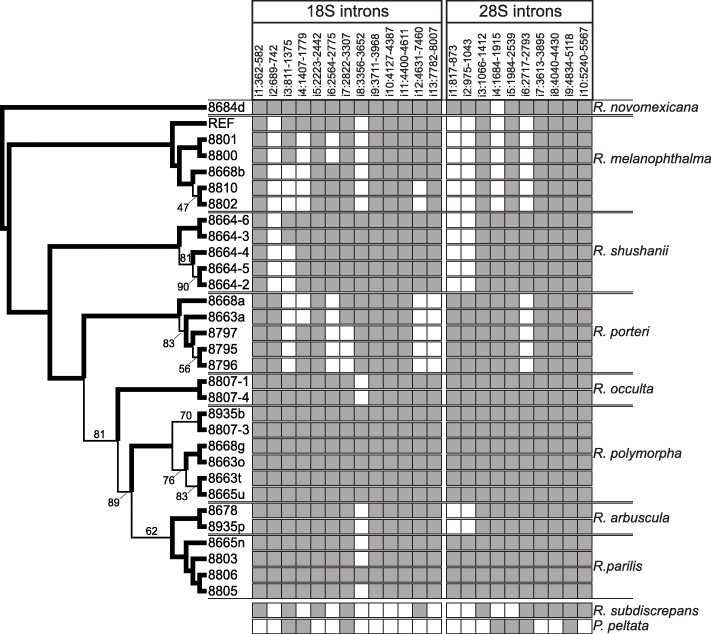
Phylogeny of the *Rhizoplaca melanophthalma* species complex inferred from the entire nuclear ribosomal cistron (18,386 nucleotide position characters) using RAxML and intron patterns in the 18S and 28S regions. Bolded branches indicate bootstrap support values (BS) ≥ 95%, and BS < 95% are indicated at nodes. The presence of introns – 13 introns in the 18S region and 10 in the 28S region – for each specimen is indicated by grey-filled squares. Presence and absence of introns is also indicated for outgroups samples – *Protoparmeliopsis peltata* and *R. subdiscrepans* (phylogenetic relationships not shown)

A total of 13 introns were identified within the 18S and ten in the 28S genes; and the intron occurrences of *Rhizoplaca* and the outgroup taxa was compared with the phylogeny inferred from the complete, aligned nrDNA region (Fig. 2).

### Phylogenetic relationships inferred from nrDNA

**Fig. 3:**
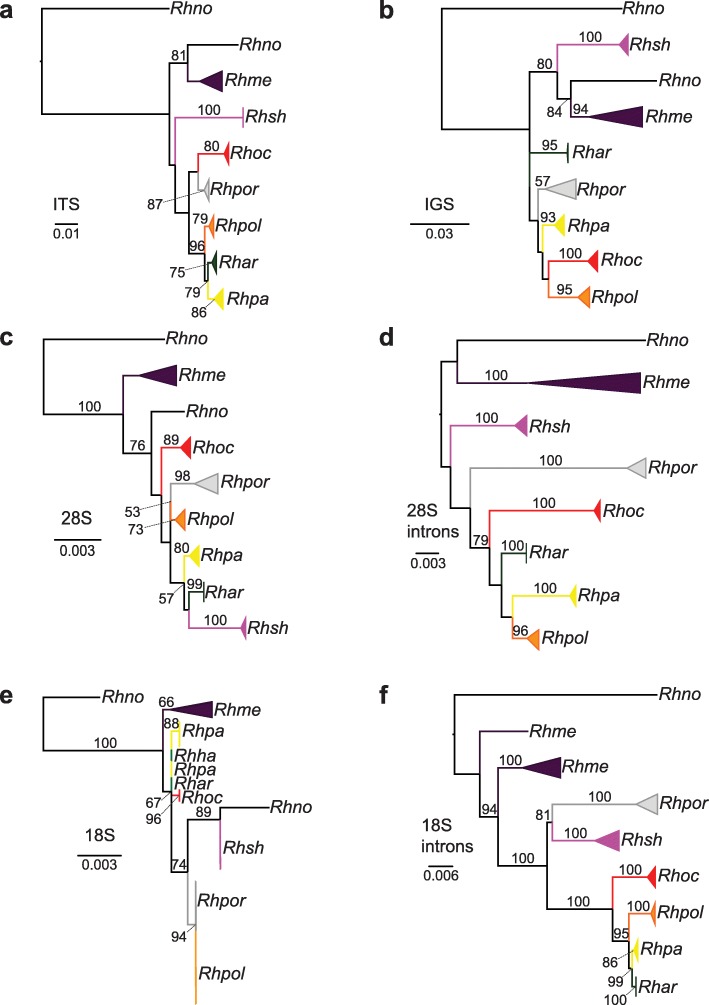
Topologies inferred from various regions of the nuclear ribosomal cistron. **a**, topology inferred from the complete ITS region (ITS1f, 5.8S, and ITS2); **b**, topology inferred from the intergenic spacer region (IGS); **c**, topology inferred from the large subunit (28S); **d**, topology inferred from introns within the 28S region; **e**, topology inferred from the small subunit region (18S); & **f**, topology inferred from introns within the 18S region. All topologies were inferred using RAxML. Tip labels: ‘*Rhar*’ = *R. arbuscula*; ‘*Rhme*’ = *R. melanophthalma*; ‘*Rhno*’ = *R. novomexicana*; ‘*Rhpa*’ = *R. parilis*; ‘*Rhpol*’ = *R. polymorpha*; ‘*Rhpor*’ = *R. porteri*; ‘*Rhoc*’ = *R. occulta*; and ‘*Rhsh*’ = *R. shushanii*

Topologies independently inferred from the ITS (ITS1, 5.8S, and ITS2), IGS, and 28S datasets independently recovered all species as monophyletic with varying levels of support among species-level lineages (Fig. 3a–c), while the topology inferred from the 18S was poorly resolved (Fig. 3e). Phylogenies inferred from the concatenated intronic regions in the 18S and 28S also recovered all species as monophyletic and generally with strong nodal support (Fig. 3d & f). Relationships among species-level clades varied widely depending on the nrDNA dataset. The maximum likelihood (ML) analyses of the complete nrDNA dataset provided a fully resolved, well-supported topology (Fig. 2).

**Fig. 4:**
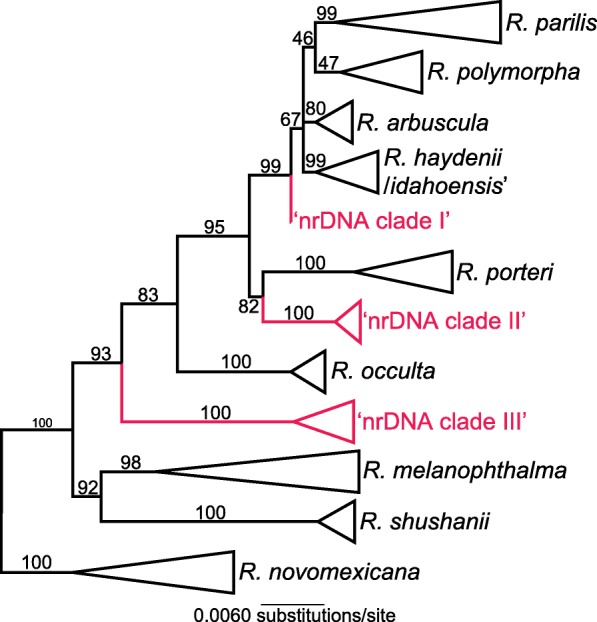
Simplified phylogeny representing broad specimen sampling (*n* = 496) and inferred from concatenated ITS and IGS sequences using IQ-tree. Bootstrap support values are indicated at nodes; and previously unknown nrDNA clades are shown in red text – “nrDNA clade I”, “nrDNA clade II”, and “nrDNA clade III” (online only). The complete phylogeny is provided as Additional file 4

In the ITS/IGS topology inferred from broad specimen sampling (*n* = 496), most previously recognized species-level clades were recovered as well-supported monophyletic clades with a few notable exceptions (Fig. 4; Additional file 4). *Rhizoplaca polymorpha* was recovered as monophyletic with weak statistical support, while *R. haydenii* sensu lato was recovered in two separate clades, one corresponding to the recently described taxon *R. arbuscula* [34] and the other comprised of specimens representing *R. haydenii* and *R. idahoensis* (Fig. 4). Three previously undetected clades were recovered in the ITS/IGS topology – ‘nrDNA clade I’, ‘nrDNA clade II’, and ‘nrDNA clade III’ (Fig. 4). Similar to other regions of nrDNA, e.g., ITS, 28S, and concatenated intron region, phylogenetic analyses of the nearly complete IGS region assembled from short read data recovered the sampled *Rhizoplaca* species as monophyletic (Fig. 3b).

## Discussion

Here we provide evidence of intragenomic copy number variation of the nrDNA operon in the *Rhizoplaca melanophthalma* species complex, ranging from nine to 43 copies. Our estimates for members of the *R. melanophthalma* species complex were in line with recent genome-based estimates for symbiotrophic fungi [7]. Given the rather limited intragenomic variation among copies (Additional file 3), it appears that specimen identification using the ITS [9] is not biased by intragenomic variation in the nrDNA region for this group [31]. While copy number variation in fungi has recently been investigated and discussed in light of our current understanding [7], below we highlight the implications of our findings as they relate to the utility of nrDNA for specimen identification, patterns of intron evolution, and the development of complete nrDNA reference libraries.

We used short reads from metagenomes of lichenized fungi to infer limited intragenomic variation in members of the *R. melanophthalma* species complex. Potentially variable sites among nrDNA operon copies did not coincide with diagnostic, fixed nucleotide position characters separating species in the *R. melanophthalma* species group (Additional file 3), providing additional evidence that distinct clades in the nrDNA topologies are not merely a reflection of variable nrDNA copies. Similarly, consistent clades are recovered from alignments of different regions of nrDNA, e.g., 28S, intergenic spacer region, and intronic regions (Fig. 4) and distinct clades in the ITS are not merely idiosyncratic in the ITS region alone.

For members of the *R. melanophthalma* species complex, we note that the distinct clades recovered from phylogenetic analyses of nrDNA do not correspond to phylogenies inferred from genome-scale data, specifically for members of the *R. porteri* group – *R. occulta*, *R. polymorpha*, and *R. porteri* [32, 33]. *Rhizoplaca occulta*, *R. polymorpha*, and *R. porteri* are recovered as divergent lineages in analyses of nrDNA, none of which are each other’s sister clade (Fig. 4), while genome-scale data consistently recover these three taxa as closely related and intermixed in a well-supported clade [32, 33]. This is in contrast to *R. melanophthalma*, *R. novomexicana, R. parilis*, and *R. shushanii* that are recovered as distinct in phylogenies inferred from both nrDNA alignments and phylogenomic data matrices. Furthermore, the present study suggests that the recently described taxon *R. arbuscula* [34] can be distinguished from the closely related clade comprising *R. haydenii* sensu stricto and *R. idahoensis* using ITS sequence data (Fig. 4; Additional file 4).

The origin of highly divergent nrDNA clades among closely related, or even conspecific, lineages remains enigmatic. The three previously undetected nrDNA clades recovered here within the *R. melanophthalma* complex, ‘nrDNA clade I’, ‘nrDNA clade II’, and ‘nrDNA clade III’ (Fig. 4), may represent previously unsampled species-level lineages, other members of the *R. porteri* clade with divergent nrDNA, or some other unexplained phenomenon. The role of hybridization/introgression has recently been proposed as an important mechanism in the diversification of the *R. melanophthalma* species complex (Keuler et al. *in review*). It is possible that divergent nrDNA clades may represent evidence of reticulate evolution with extinct or as-of-yet unsampled species or simply artifacts of hybridization/introgression. However, additional broader sampling will be required to infer the relationship of these newly found divergent nrDNA lineages. Similarly, the number and diversity of lichen associated symbionts has recently been shown to be far more complex than initially thought [35]. These studies suggest that the dynamics of symbiotic genetics may be synergistic with intricate interactions that combine to support these unique and complicated symbiotic systems.

With the use of additional high-throughput sequencing methods, including single molecule sequencing and/or long-read sequencing technologies it will be possible to more directly characterize intragenomic variation and copy number of nrDNA [1, 36, 37]. In this study, we demonstrate the potential for intraspecific copy number variation for all *Rhizoplaca* species represented by > 1 specimen, including conspecific specimens collected at close spatial scales (Table 1). Recent studies provide novel insights into the ploidy level of lichen-forming fungi, which are traditionally considered to be haploid. However, future research will be required to confirm that vegetative *Rhizoplaca* thalli are, in fact, consistently haploid or if more complex scenarios of ploidy levels occur in lichen thalli [38, 39].

Here, intragenomic variation in the nrDNA operon was observed to be low in the *R. melanophthalma* species complex. Concerted evolution is considered to be responsible for the maintenance of sequence similarity among copies of nrDNA repeats in the operon potentially leading to low intragenomic variation [40, 41]. Concerted evolution is driven by unequal crossing over and gene conversion and although these two processes both lead to the homogenization of nrDNA repeat units, their underlying mechanisms differ from each other [41, 42]. Gene conversion is caused by replacement of one DNA sequence by another in a unidirectional manner during homologous recombination. Unequal crossing over, on the other hand, involves the misalignment of homologous chromosomes in meiosis or sister chromatids in mitosis followed by nonreciprocal transfer of DNA sequence from one chromosomal location to another resulting in a deletion in one of the chromosomes and a duplication in the other. While both these mechanisms lead to homogenization of nrDNA repeat units, unequal crossing over also has the potential to affect the copy number of the repeat units [40, 43].

The results of this study revealed striking patterns of multiple intron gains and losses in the *R. melanophthalma* group. Eleven of the 23 introns found in the 18S and 28S nrDNA genes are present in all *Rhizoplaca* specimens (i1, i5, i9, i10, i11 in the 18S gene and i3, i5, i7 to i10 in the 28S gene), but the pattern of all other introns is highly variable indicating multiple gains and losses in the evolution of *Rhizoplaca*. When comparing the intron pattern with the phylogenetic tree, only the occurrence of three introns can be most parsimoniously explained by a single event, i. e. the loss i7 from the 18S gene in some *R. porteri*, the loss of i13 from the 18S gene in all *R. porteri,* and a gain of i4 in the 28S gene after the split of *R. novomexicana* and *R. melanophthalma* from the remaining *Rhizoplaca*. All other introns would require at least two (i2, i3, i4, i6, i12 in 18S and i1, i2, i6 in 28S) or four losses (i8 in 18S) to reassemble the intron pattern. This intron pattern confirms that nrDNA introns are highly mobile and the dynamic variability of the nrDNA region [44].

## Conclusions

The results from our study support the general assumption that the ITS region of the nrDNA is an effective barcoding marker for fungi. The recent development of general primers that allow for amplification of the complete ribosomal operon, in conjunction with PacBio and Nanopore sequencing technologies, have helped promote the development of more comprehensive nrDNA databases [1]. The nearly complete ribosomal operons assembled for this study rank among the first generated for lichen-forming fungi. These data span highly conservative nrDNA regions that are important for high-level taxonomic classification to highly variable regions that can serve for population-level inference. A number of questions related to nrDNA evolution remain unanswered, including processes driving concerted evolution [40], copy number variation – particularly in symbiotic fungi [7], and patterns of intron distribution [4, 44, 45].

## Methods

### Taxonomic sampling and data compilation

Our sampling included representatives of eight formally recognized species within the *R. melanophthalma* species complex [31] and two outgroup taxa – *R. subdiscrepans* (Nyl.) R. Sant*.* and *Protoparmeliopsis peltata* (Ramond) Arup, Zhao Xin & Lumbsch (Additional file 1). For this study, we analyzed short-read metagenomic data from a total of 33 specimens, representing ten *Rhizoplaca* s. lat. Species (Leavitt et al., 2016), including: *R. arbuscula* Rosentreter, St. Clair & Leavitt (*n* = 2), *R. melanophthalma* (DC.) Leuckert (*n* = 6), *R. novomexicana *(H. Magn.) S.D. Leav., Zhao Xin & Lumbsch (*n* = 1), *R. occulta* S.D. Leav., Fern-Mend., Lumbsch, Sohrabi & St. Clair (*n* = 2), *R. parilis* S.D. Leav., Fern.-Mend., Lumbsch, Sohrabi & St. Clair (*n* = 4), *R. polymorpha* S.D. Leav., Fern.-Mend., Lumbsch, Sohrabi & St. Clair (*n* = 6), *R. porteri* S.D. Leav., Fern.-Mend., Lumbsch, Sohrabi & St. Clair (*n* = 5), *R. shushanii* S.D. Leav., Fern.-Mend., Lumbsch, Sohrabi & St. Clair (*n* = 5), and single representatives of two outgroup taxa – *R. subdiscrepans* and *P. peltata*. Initial identifications were based on morphology and DNA barcode identification using the ITS region [31]. All specimens used in this study are housed at the Herbarium of Non-Vascular Cryptogams (BRY-C) at Brigham Young University.

In order to more fully characterize the range of ITS diversity in the *Rhizoplaca melanophthalma* species complex, amplicon-based sequence data was generated for a total of 496 specimens from the *R. melanophthalma* species complex collected from various sites throughout western North America, the center of species diversity for this group [30]. For all new specimens, DNA was extracted using the Wizard Genomic DNA Purification Kit (Promega), and amplification and sequencing of the ITS marker followed previously described methods [29]. Newly generated sequences were combined with previously available nrDNA sequence data from the *Rhizoplaca melanophthalma* group (https://treebase.org/; study No. 19048).

### Short-read data, genome assembly, and identification of the nuclear ribosomal operon

Short reads from 33 *Rhizoplaca* specimens reported in a previous study [32] were used for genome assembly to identify contigs containing the nuclear ribosomal operon (deposited in NCBI’s Sequence Read Archive under project PRJNA576709). Full details of specimen preparation and sequencing are described in [32]. In short, DNA was extracted from a small fragment of each lichen thallus (comprised of multiple lichen symbionts, including the targeted mycobiont), libraries were prepared using the Illumina Nextera XT DNA library prep kit (product discontinued), then pooled and sequenced using a single lane on the Illumina HiSeq2000 platform, generating 100-bp paired-end reads with a 350-bp insert size. Four specimens were sequenced individually on the MiSeq platform (Illumina) generating 250-bp paired-end (PE) reads with a 550-bp insert size. While reads from the axenic reference culture (‘mela_REF’) were exclusively derived from the targeted *R. melanophthalma* fungal genome, genomic libraries prepared from all field-collected specimens were comprised not only of DNA from the targeted mycobiont but also DNA from the complete holobiont, e.g., associated *Trebouxia* photobiont, secondary fungi, bacteria, etc. [46–50].

All paired-end (PE) reads were filtered using Trimmomatic v0.33 [51] before assembly to remove low-quality reads and/or included contamination from Illumina adaptors using the following parameters: ILLUMINACLIP; LEADING:3; TRAILING:3; SLIDINGWINDOW:4:15; and MINLEN:36. De novo genome assemblies were constructed using SPAdes v3.5.0 [52] while running a single read error correction iteration prior to the genome assembly using k-mer values of 55, 77, 99, with the mismatch careful mode (−-careful) enabled. From each assembly, contigs containing the nrDNA were identified using a custom BLAST [53] search implemented in the program Geneious R11 [54] against available regions of the nrDNA, e.g., 28S, IGS, and ITS, generated from *Rhizoplaca melanophthalma* s. lat. Specimens.

Some regions of the nrDNA operon are highly conserved across divergent lineages (e.g. 18S, 5.8S, and portions of 28S subunit), and reads from non-target genomes (e.g., the photobiont, accessory fungi) may potentially bias the interpretation of intragenomic variation within *Rhizoplaca* species. Therefore, we used a de novo assembly approach for all nrDNA reads in order to separate nrDNA cluster of the targeted *Rhizoplaca* mycobiont from reads of other symbionts that co-occur within lichens. For each specimen, PE reads were mapped back to the respective contigs containing nrDNA from the SPAdes assembly using the Geneious R11 Read Mapper, with the “medium-low sensitivity/ fast” settings, iterated 5 times. Successfully mapped reads were then assembled de novo using the native Geneious R11 Assembler using the ‘medium-low sensitivity’ parameter. Resulting contigs were searched against NCBI’s GenBank database [55] using BLAST [56] to identify non-target contigs, which were then excluded from further analysis.

### Assessing intragenomic variation of the ITS, inferring copy number of the ribosomal operon, and intron identification

Our assessment of potential intragenomic variation focused on the ITS region (ITS1, 5.8S, and ITS2) [9]. To identify potentially polymorphic sites in the nrDNA, PE reads from each specimen were mapped back to their corresponding ITS region extracted from the Geneious assembly, with a 600 bp buffer on either end, using BWA [57]. The 600 bp buffer on either end was used to ensure that all reads containing portions of the ITS region were indeed mapped back to the reference rather than being discarded because part of the read mapped to a region of the ribosomal operon before or after the ITS region. The Samtools v1.6 genomics utilities package [58] was used to process alignment output, filtering out unmapped reads so that only reads corresponding to the ITS and bordering regions remained. A samtools pileup file was then generated to identify the bases aligned with each position of the reference sequence which was visually confirmed using Geneious v11. Custom R scripts were used to identify mismatches and calculate percent variance at each position in the pileup file (available on GitHub: https://github.com/MSBradshaw/LichenBarcoding). To calculate percent variance, the number of reads that varied from the consensus at each nucleotide position character was divided by coverage at that location. When calculating percent variance, no effort was made to identify bases within a read that were the result of sequencing error versus true variation, e.g., sequencing error and true variation would be distinguishable based on the percent variance. Sequencing error would be comparable to known error rates of the sequencing technology, ≤0.1% is achieved for ≥75–85% of base [59], while true intragenomic variation would exceed the error rate for Illumina sequencing.

To estimate the total copy number of the nrDNA operon, we compared average read depth coverage of the nrDNA relative to coverage of putative single-copy regions of the nuclear genome [7]. For each specimen, reads were mapped back to their respective Geneious assembly of the nrDNA operon, the three largest contigs (ca. 363 kb, 307 kb, and 227 kb, respectively) from the draft genome assembly from the axenic culture [32], and three known single-copy genes, *MCM7*, *RPB1* and *RPB2*. The average coverage depth of the nrDNA operon was divided by the average coverage depth of the nuclear single copy genes and nuclear genomic regions. The difference in coverage was interpreted as an approximation of the copy number of the nrDNA operon.

We used Rfam models to demark boundaries of the 18S, 5.8S, and 28S regions and to identify introns [60]. We used an annotated nrDNA sequence from *Saccharomyces paradoxus* (GenBank accession No. BR000309) to corroborate boundaries between the 18S, ITS1, 5.8S, ITS, 28S, and IGS regions and introns inferred using Rfam. In contrast to most other eukaryotic genomes, yeast genomes have few introns [61] and the highly conserved 18S & 28S regions in *S. paradoxux* can help demark boundaries among nrDNA regions. A multiple sequence alignment (MSA) of the nearly complete nrDNA operon assemblies from the *Rhizoplaca* specimens and the *S. paradoxux* sequence was performed using the program MAFFT v7 [62, 63], implementing the FFT-NS-i iterative refinement method. No attempt was made to distinguish different intron types – e.g., group I, group II, and spliceosomal introns.

A group I intron at the 3′ end of the 18S has previously been shown to be present in all species within the *R. melanophthalma* group, except *R. porteri* [29]; and the absence of this intron serves as a diagnostic character in the description of this taxon [31]. However, PCR amplifications may not provide an accurate assessment of repetitive genomic regions due to PCR bias or an overwhelming signal from the most commonly amplified variant. Therefore, to verify the absence of this group I intron, we attempted to map reads from *R. porteri* specimens to a consensus sequence representing this intron with the Geneious v11 Read Mapper, using the “medium-low sensitivity/ fast” settings, iterated 5 times. To test if this group I intron might be absent in some copies of nrDNA found in other species in the *R. melanophthalma* group, we searched PE reads from all *R. arbuscula*, *R. melanophthalma*, *R. parilis*, *R. polymorpha*, *R. porteri*, *R. occulta*, and *R. shushanii* specimens for the conserved motif lacking the intron using a custom script.

### Multiple sequence alignments and phylogenetic inferences

An initial MSA of the nearly complete nrDNA operon assemblies from the *Rhizoplaca* specimens (*n* = 33) was performed using the program MAFFT v7 [62, 63], implementing the FFT-NS-i iterative refinement method. To improve alignment accuracy for specific phylogenetic comparisons of different regions within the ribosomal operon, individual alignments were constructed independently for the 18S, ITS (ITS1, 5.8S, ITS2), 28S, the IGS, and each intron present in the 18S and 28S regions. After excluding introns, MSAs of the 18S, 28S nrDNA, and ITS region were aligned in MAFFT using the G-INS-i algorithm. The IGS and intronic regions were aligned individually using the E-INS-i algorithm for sequences with conserved domains and long gaps.

Previous studies have indicated that species in the *R. melanophthalma* species complex can be distinguished using phylogenetic inferences based on the standard barcoding marker for fungi [29, 31]. Here we also investigated the question as to whether or not species within this complex can be recovered as monophyletic using other regions of nrDNA for phylogenetic inference. We inferred phylogenies from different regions of the ribosomal operon: (i) 18S nrDNA, excluding introns; (ii) introns within the 18S region; (iii) 28S nrDNA, excluding introns; (iv) introns within the 28S region; (v) concatenated 18S and 28S nrDNA, excluding introns; (vi) concatenated introns from both 18S and 28S regions, (vii) the IGS region; and (viii) a complete matrix comprised of 18S and 28S nrDNA, and associated introns, and the IGS region. Only introns that were present in all of the ingroup samples – the *R. melanophthalma* group – were included in phylogenetic analyses to minimize bias from highly mobile introns that may have been incorporated or lost more recently than the most recent common ancestor of the *R. melanophthalma* group.

ML topologies were inferred individually from each of these regions individually using the program RAxML v8.2.2 [64]. All ML analyses were performed using the CIPRES Science Gateway server (http://www.phylo.org/portal2/), using the ‘GTRGAMMA’ model and evaluating nodal support using 1000 bootstrap pseudo-replicates. A ML topology was also inferred from the complete nrDNA matrix using RAxML, treating each region (IGS, 18S [including associated introns], ITS, and 28S [including associated introns]) as separate partitions; otherwise analyses were performed as described above.

To compare the nrDNA sequence variation of the 33 specimens sampled from the *R. melanophthalma* group within the context of a broader sampling of specimens, we compiled a nrDNA matrix comprised of IGS and ITS sequences from a previous study [65] with our newly sampled specimens, resulting in a total of 496 specimens (https://treebase.org/; study No. 24225). For comparison, a number of specimens were represented by multiple ITS sequences, including those assembled from PE reads for this study and sequences generated using Sanger sequencing from the initial DNA extractions used for high-throughput sequencing library preparation. A ML topology was inferred from this larger dataset using IQ-tree v1.6.3 [66], treating each region (IGS and ITS) as separate partitions, with 1000 ultra-fast bootstrap replicates [67] to assess nodal support, and the best-fit substitution models as predicted by ModelFinder [68].

## Supplementary information

**Additional file 1.** List of sampled specimens, DNA code, locality, number of filtered reads, length of assembled nrDNA cistron, and average coverage

**Additional file 2. **Detailed list of intragenomic variation in specimens representing seven species the *R. melanophthalma* species complex. The inferred nucleotide is indicated, the read count for each nucleotide at potentially variable positions in the ITS sequence, and the estimated variance

**Additional file 3. **Intragenomic variation in specimens representing seven species the *R. melanophthalma* species complex. Each specimen is represented by two rows: the top row indicates the level of variance of each aligned nucleotide position character, separated into four groups – (i) > 10% variation, (ii) 1–9%, (iii) < 1% but > than 0, and (iv) no variance; the lower row indicates polymorphic sites in the multiple sequence alignment of the ITS region

**Additional file 4. **Phylogeny inferred from concatenated ITS and IGS sequences that were generated from a broad sampling (*n* = 496) of specimens from the *Rhizoplaca melanophthalma* species complex. Bootstrap support values are indicated at nodes. Species-level clades are highlighted with distinct colors

## Data Availability

Short reads are deposited in NCBI’s Sequence Read Archive under project PRJNA576709; and assembled sequences are deposited in GenBank (accession numbers MN756803– MN756835; MN764257-MN764288).

## Abbreviations

BRY-C: Herbarium of Non-Vascular Cryptogams, Brigham Young University
BS: Bootstrap
IGS: Intergenic spacer region
ITS: Internal transcribed spacer region
ML: Maximum likelihood
MSA: Multiple sequence alignment
NCBI: National center for biotechnology information
nrDNA: Nuclear ribosomal DNA
PCR: Polymerase chain reaction
PE: Paired end
SD: Standard deviation

## References

[1] Wurzbacher C, Larsson E, Bengtsson-Palme J, Van den Wyngaert S, Svantesson S, Kristiansson E, Kagami M, Nilsson RH (2019). Introducing ribosomal tandem repeat barcoding for fungi. Mol Ecol Resour.

[2] White T.J., Bruns T., Lee S., Taylor J. (1990). AMPLIFICATION AND DIRECT SEQUENCING OF FUNGAL RIBOSOMAL RNA GENES FOR PHYLOGENETICS. PCR Protocols.

[3] Hillis DM, Dixon MT (1991). Ribosomal DNA. Molecular evolution and phylogenetic inference. Q Rev Biol.

[4] Gutiérrez G, Blanco O, Divakar P, Lumbsch H, Crespo A (2007). Patterns of group I intron presence in nuclear SSU rDNA of the lichen family Parmeliaceae. J Mol Evol.

[5] Alm Rosenblad M, Martín MP, Tedersoo L, Ryberg MK, Larsson E, Wurzbacher C, Abarenkov K, Nilsson RH (2016). Detection of signal recognition particle (SRP) RNAs in the nuclear ribosomal internal transcribed spacer 1 (ITS1) of three lineages of ectomycorrhizal fungi (Agaricomycetes, Basidiomycota). MycoKeys.

[6] Szostak JW, Wu R (1980). Unequal crossing over in the ribosomal DNA of *Saccharomyces cerevisiae*. Nature..

[7] Lofgren LA, Uehling JK, Branco S, Bruns TD, Martin F, Kennedy PG (2019). Genome-based estimates of fungal rDNA copy number variation across phylogenetic scales and ecological lifestyles. Mol Ecol.

[8] Lindner DL, Banik MT (2011). Intragenomic variation in the ITS rDNA region obscures phylogenetic relationships and inflates estimates of operational taxonomic units in genus *Laetiporus*. Mycologia..

[9] Schoch CL, Seifert KA, Huhndorf S, Robert V, Spouge JL, Levesque CA, Chen W (2012). Nuclear ribosomal internal transcribed spacer (ITS) region as a universal DNA barcode marker for Fungi. Proc Natl Acad Sci U S A.

[10] Nilsson RH, Larsson K-H, Taylor AFS, Bengtsson-Palme J, Jeppesen TS, Schigel D, Kennedy P, Picard K, Glöckner FO, Tedersoo L (2019). The UNITE database for molecular identification of fungi: handling dark taxa and parallel taxonomic classifications. Nucleic Acids Res.

[11] Ganley ARD, Kobayashi T (2007). Highly efficient concerted evolution in the ribosomal DNA repeats: Total rDNA repeat variation revealed by whole-genome shotgun sequence data. Genome Res.

[12] Simon UK, Weiß M (2008). Intragenomic variation of fungal ribosomal genes is higher than previously thought. Mol Biol Evol.

[13] Green SJ, Venkatramanan R, Naqib A (2015). Deconstructing the polymerase chain reaction: understanding and correcting bias associated with primer degeneracies and primer-template mismatches. PLoS One.

[14] Harrington TC, Kazmi MR, Al-Sadi AM, Ismail SI (2014). Intraspecific and intragenomic variability of ITS rDNA sequences reveals taxonomic problems in *Ceratocystis fimbriata* sensu stricto. Mycologia..

[15] Lindner DL, Carlsen T, Henrik Nilsson R, Davey M, Schumacher T, Kauserud H (2013). Employing 454 amplicon pyrosequencing to reveal intragenomic divergence in the internal transcribed spacer rDNA region in fungi. Ecol Evol.

[16] Mark K, Cornejo C, Keller C, Flück D, Scheidegger C (2016). Barcoding lichen-forming fungi using 454 pyrosequencing is challenged by artifactual and biological sequence variation. Genome..

[17] Myllys L, Lohtander K, Källersjö M, Tehler A (1999). Sequence insertions and ITS data provide congruent information on *Roccella canariensis* and *R. tuberculata* (Arthoniales, Euascomycetes) phylogeny. Mol Phylogenet Evol.

[18] Thell A (1999). Group I intron versus ITS sequences in phylogeny of Cetrarioid lichens. Lichenologist.

[19] Gargas A, DePriest PT, Grube M, Tehler A (1995). Multiple origins of lichen symbioses in fungi suggested by SSU rDNA phylogeny. Sci.

[20] DePriest PT (1993). Molecular innovations in lichen systematics: the use of ribosomal and intron nucleotide sequences in the *Cladonia chlorophaea* complex. Bryologist.

[21] Pino-Bodas R, Martin AP, Burgaz AR, Lumbsch HT (2013). Species delimitation in *Cladonia* (Ascomycota): a challenge to the DNA barcoding philosophy. Mol Ecol Resour.

[22] Sadowska-Deś AD, Bálint M, Otte J, Schmitt I (2013). Assessing intraspecific diversity in a lichen-forming fungus and its green algal symbiont: evaluation of eight molecular markers. Fungal Ecol.

[23] Leavitt SD, Esslinger TL, Hansen ES, Divakar PK, Crespo A, Loomis BF, Lumbsch HT (2014). DNA barcoding of brown Parmeliae (Parmeliaceae) species: a molecular approach for accurate specimen identification, emphasizing species in Greenland. Org Divers Evol.

[24] Kelly LJ, Hollingsworth PM, Coppins BJ, Ellis CJ, Harrold P, Tosh J, Yahr R (2011). DNA barcoding of lichenized fungi demonstrates high identification success in a floristic context. New Phytol.

[25] Divakar PK, Leavitt SD, Molina MC, Del-Prado R, Lumbsch HT, Crespo A (2016). A DNA barcoding approach for identification of hidden diversity in Parmeliaceae (Ascomycota): *Parmelia sensu* stricto as a case study. Bot J Linn Soc.

[26] Kanz B (2015). Brackel Wv, Cezanne R, Eichler M, Hohmann M-L, Teuber D, Printzen C. DNA barcodes for the distinction of reindeer lichens: a case study using *Cladonia rangiferina* and *C. stygia*. Herzogia.

[27] Yahr R, Schoch Conrad L, Dentinger Bryn TM (2016). Scaling up discovery of hidden diversity in fungi: impacts of barcoding approaches. Philos Trans Royal Soc B.

[28] Simon DM, Hummel CL, Sheeley SL, Bhattacharya D (2005). Heterogeneity of intron presence or absence in rDNA genes of the lichen species *Physcia aipolia* and *P. stellaris*. Curr Genet.

[29] Leavitt SD, Fankhauser JD, Leavitt DH, Porter LD, Johnson LA, St Clair LL (2011). Complex patterns of speciation in cosmopolitan “rock posy” lichens – discovering and delimiting cryptic fungal species in the lichen-forming *Rhizoplaca melanophthalma* species-complex (Lecanoraceae, Ascomycota). Mol Phylogenetics Evol.

[30] Leavitt SD, Fernández-Mendoza F, Pérez-Ortega S, Sohrabi M, Divakar PK, Vondrák J, Thorsten Lumbsch H, St Clair LL (2013). Local representation of global diversity in a cosmopolitan lichen-forming fungal species complex (*Rhizoplaca*, Ascomycota). J Biogeogr.

[31] Leavitt SD, Fernández-Mendoza F, Pérez-Ortega S, Sohrabi M, Divakar PK, Lumbsch HT, St Clair LL (2013). DNA barcode identification of lichen-forming fungal species in the *Rhizoplaca melanophthalma* species-complex (Lecanorales, Lecanoraceae), including five new species. MycoKeys.

[32] Leavitt SD, Grewe F, Widhelm T, Muggia L, Wray B, Lumbsch HT (2016). Resolving evolutionary relationships in lichen-forming fungi using diverse phylogenomic datasets and analytical approaches. Sci Rep.

[33] Grewe F, Huang J-P, Leavitt SD, Lumbsch HT (2017). Reference-based RADseq resolves robust relationships among closely related species of lichen-forming fungi using metagenomic DNA. Sci Rep.

[34] Leavitt Steven D., Keuler Rachel, Newberry Clayton C., Rosentreter Roger, Clair Larry L. St. (2019). Shotgun sequencing decades-old lichen specimens to resolve phylogenomic placement of type material. Plant and Fungal Systematics.

[35] Spribille T (2018). Relative symbiont input and the lichen symbiotic outcome. Curr Opin Plant Biol.

[36] English AC, Richards S, Han Y, Wang M, Vee V, Qu J, Qin X, Muzny DM, Reid JG, Worley KC (2012). Mind the gap: upgrading genomes with Pacific biosciences RS long-read sequencing technology. PLoS One.

[37] Clarke J, Wu H-C, Jayasinghe L, Patel A, Reid S, Bayley H (2009). Continuous base identification for single-molecule nanopore DNA sequencing. Nat Nanotechnol.

[38] Tripp EA, Zhuang Y, Lendemer JC (2017). A review of existing whole genome data suggests lichen mycelia may be haploid or diploid. Bryologist.

[39] Pizarro D, Dal Grande F, Leavitt SD, Dyer PS, Schmitt I, Crespo A, Thorsten Lumbsch H, Divakar PK (2019). Whole-genome sequence data uncover widespread heterothallism in the largest group of lichen-forming fungi. Genome Biol Evol.

[40] Nei M, Rooney AP (2005). Concerted and birth-and-death evolution of multigene families. Annu Rev Genet.

[41] Eickbush TH, Eickbush DG (2007). Finely orchestrated movements: evolution of the ribosomal RNA genes. Genet.

[42] Naidoo K, Steenkamp ET, Coetzee MPA, Wingfield MJ, Wingfield BD (2013). Concerted evolution in the ribosomal RNA Cistron. PLoS One.

[43] Pinhal D, Yoshimura TS, Araki CS, Martins C (2011). The 5S rDNA family evolves through concerted and birth-and-death evolution in fish genomes: an example from freshwater stingrays. BMC Evol Biol.

[44] DePriest PT, Been MD (1992). Numerous group I introns with variable distributions in the ribosomal DNA of a lichen fungus. J Mol Biol.

[45] Hibbett DS (1996). Phylogenetic evidence for horizontal transmission of group I introns in the nuclear ribosomal DNA of mushroom-forming fungi. Mol Biol Evol.

[46] Arnold AE, Miadlikowska J, Higgins KL, Sarvate SD, Gugger P, Way A, Hofstetter V, Kauff F, Lutzoni F (2009). A phylogenetic estimation of trophic transition networks for ascomycetous fungi: are lichens cradles of symbiotrophic fungal diversification?. Syst Biol.

[47] Cardinale M, JVdC MH, Berg G, Grube M (2008). In situ analysis of the bacterial community associated with the reindeer lichen Cladonia arbuscula reveals predominance of Alphaproteobacteria. FEMS Microbiol Ecol.

[48] Grube M, Cardinale M, de Castro JV, Muller H, Berg G (2009). Species-specific structural and functional diversity of bacterial communities in lichen symbioses. ISME J.

[49] Hodkinson B, Lutzoni F (2009). A microbiotic survey of lichen-associated bacteria reveals a new lineage from the Rhizobiales. Symbiosis.

[50] Muggia L, Vancurova L, Škaloud P, Peksa O, Wedin M, Grube M (2013). The symbiotic playground of lichen thalli – a highly flexible photobiont association in rock-inhabiting lichens. FEMS Microbiol Ecol.

[51] Bolger AM, Lohse M, Usadel B (2014). Trimmomatic: a flexible trimmer for Illumina sequence data. Bioinformatics.

[52] Nurk S, Bankevich A, Antipov D, Gurevich A, Korobeynikov A, Lapidus A, Prjibelsky A, Pyshkin A, Sirotkin A, Sirotkin Y *et al*. Assembling genomes and mini-metagenomes from highly chimeric reads. In: Deng M, Jiang R, Sun F, Zhang X, editors. Res Comput Mol Biol*.* vol. 7821: Berlin: Springer Berlin Heidelberg; 2013. p. 158–170.

[53] Altschul SF, Gish W, Miller W, Myers EW, Lipman DJ (1990). Basic local alignment search tool. J Mol Biol.

[54] Kearse M, Moir R, Wilson A, Stones-Havas S, Cheung M, Sturrock S, Buxton S, Cooper A, Markowitz S, Duran C (2012). Geneious basic: an integrated and extendable desktop software platform for the organization and analysis of sequence data. Bioinform.

[55] Benson DA, Cavanaugh M, Clark K, Karsch-Mizrachi I, Ostell J, Pruitt KD, Sayers EW (2018). GenBank. Nucleic Acids Res.

[56] Altschul SF, Madden TL, Schaffer AA, Zhang J, Zhang Z, Miller W, Lipman DJ (1997). Gapped BLAST and PSI-BLAST: a new generation of protein database search programs. Nucleic Acids Res.

[57] Li H, Durbin R (2010). Fast and accurate long-read alignment with burrows–wheeler transform. Bioinform.

[58] Li H, Handsaker B, Wysoker A, Fennell T, Ruan J, Homer N, Marth G, Abecasis G, Durbin R, Subgroup GPDP (2009). The sequence alignment/map format and SAMtools. Bioinform.

[59] Ross MG, Russ C, Costello M, Hollinger A, Lennon NJ, Hegarty R, Nusbaum C, Jaffe DB (2013). Characterizing and measuring bias in sequence data. Genome Biol.

[60] Kalvari I, Argasinska J, Quinones-Olvera N, Nawrocki EP, Rivas E, Eddy SR, Bateman A, Finn RD, Petrov AI (2017). Rfam 13.0: shifting to a genome-centric resource for non-coding RNA families. Nucleic Acids Res.

[61] Spingola M, Grate L, Haussler D, Ares M (1999). Genome-wide bioinformatic and molecular analysis of introns in *Saccharomyces cerevisiae*. RNA.

[62] Katoh K, Kuma K-I, Toh H, Miyata T (2005). MAFFT version 5: improvement in accuracy of multiple sequence alignment. Nucleic Acids Res.

[63] Katoh K, Toh H (2008). Recent developments in the MAFFT multiple sequence alignment program. Brief Bioinform.

[64] Stamatakis A (2014). RAxML version 8: a tool for phylogenetic analysis and post-analysis of large phylogenies. Bioinform.

[65] Leavitt SD, Kraichak E, Vondrak J, Nelsen MP, Sohrabi M, Perez-Ortega S, St Clair LL, Lumbsch HT (2016). Cryptic diversity and symbiont interactions in rock-posy lichens. Mol Phylogenetics Evol.

[66] Nguyen L-T, Schmidt HA, von Haeseler A, Minh BQ (2014). IQ-TREE: a fast and effective stochastic algorithm for estimating maximum-likelihood phylogenies. Mol Biol Evol.

[67] Hoang DT, Chernomor O, von Haeseler A, Minh BQ, Vinh LS (2018). UFBoot2. Improving the ultrafast bootstrap approximation. Mol Biol Evol.

[68] Kalyaanamoorthy S, Minh BQ, Wong TKF, von Haeseler A, Jermiin LS (2017). ModelFinder: fast model selection for accurate phylogenetic estimates. Nat Methods.

