# Supplementary figures and images for: Characterizing the ribosomal tandem repeat and its utility as a DNA barcode in lichen-forming fungi
Source: BMC Evol Biol. 2020 Jan 6;20:2. doi: 10.1186/s12862-019-1571-4 (PMC6945747; doi:10.1186/s12862-019-1571-4)

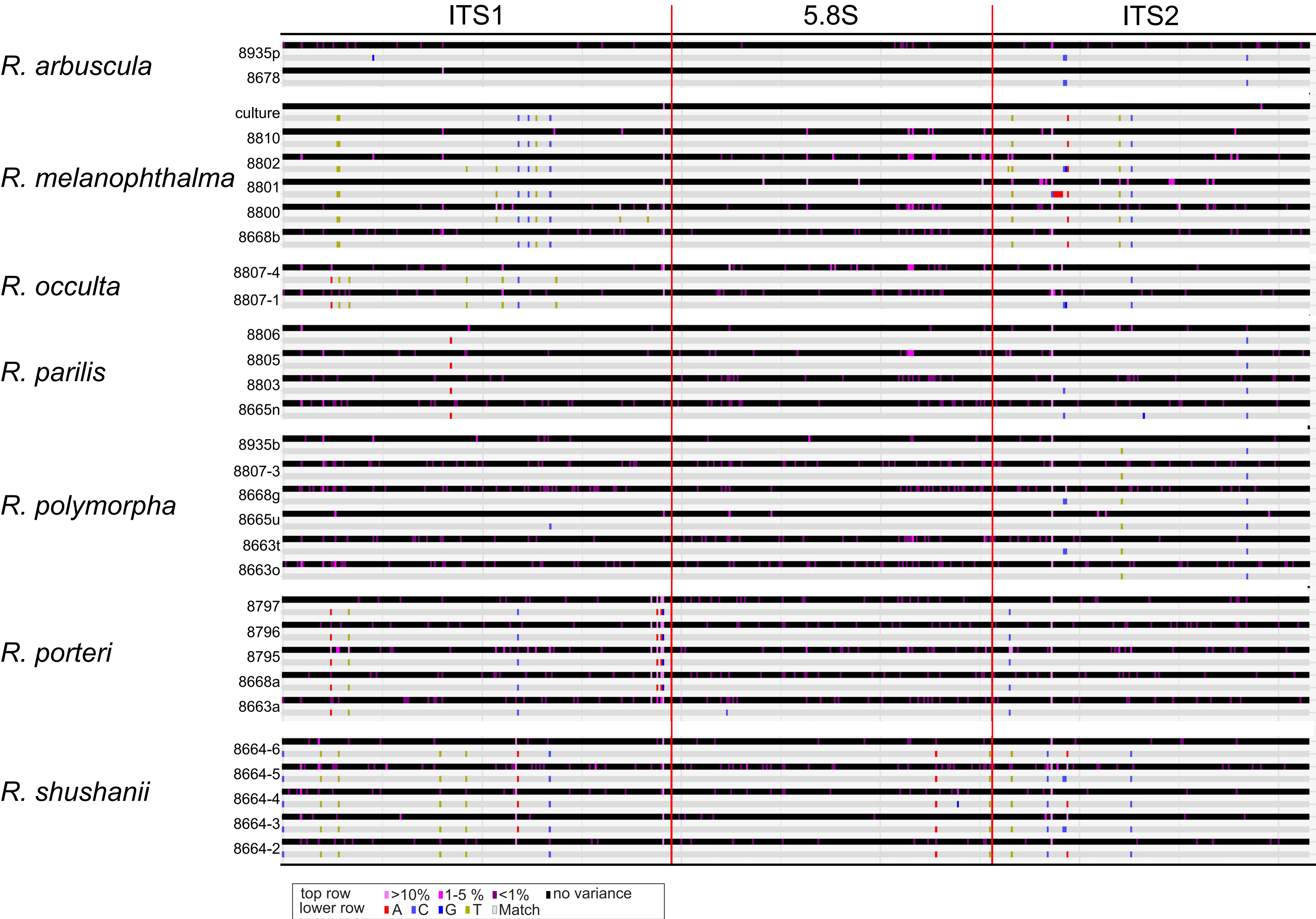

Supplement: Supplementary file 3 — Additional file 3. Intragenomic variation in specimens representing seven species the R. melanophthalma species complex. Each specimen is represented by two rows: the top row indicates the level of variance of each aligned nucleotide position character, separated into four groups – (i) > 10% variation, (ii) 1–9%, (iii) < 1% but > than 0, and (iv) no variance; the lower row indicates polymorphic sites in the multiple sequence alignment of the ITS region [file 12862_2019_1571_MOESM3_ESM.pdf]

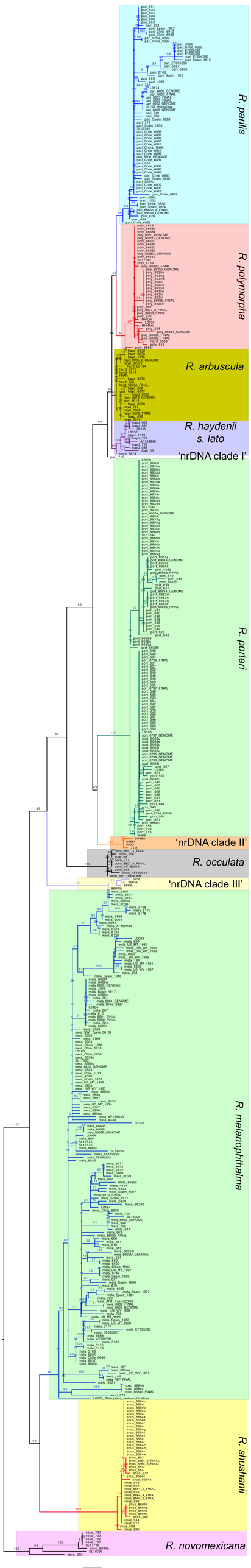

Supplement: Supplementary file 4 — Additional file 4. Phylogeny inferred from concatenated ITS and IGS sequences that were generated from a broad sampling (n = 496) of specimens from the Rhizoplaca melanophthalma species complex. Bootstrap support values are indicated at nodes. Species-level clades are highlighted with distinct colors [file 12862_2019_1571_MOESM4_ESM.pdf]
